# 2-Amino-5-chloro­pyridinium 4-carb­oxy­butano­ate

**DOI:** 10.1107/S1600536810027091

**Published:** 2010-07-14

**Authors:** Madhukar Hemamalini, Hoong-Kun Fun

**Affiliations:** a X-ray Crystallography Unit, School of Physics, Universiti Sains Malaysia, 11800 USM, Penang, Malaysia

## Abstract

In the title salt, C_5_H_6_ClN_2_^+^·C_5_H_7_O_4_^−^, the 2-amino-5-chloro­pyridinium cation is essentially planar, with a maximum deviation of 0.010 (3) Å. In the crystal structure, the protonated N atom and the 2-amino group of the cation are hydrogen bonded to the carboxyl­ate O atoms of the anion *via* a pair of N—H⋯O hydrogen bonds, forming an *R*_2_^2^(8) ring motif. The ion pairs are further connected *via* O—H⋯O, N—H⋯O and C—H⋯O hydrogen bonds, forming a layer parallel to the *bc* plane. In the layer, the hydrogen glutarate anions self-assemble *via* O—H⋯O hydrogen bonds, forming a supra­molecular chain along the *c* axis. Furthermore, the cations and anions are stacked down along the *a* axis, forming a three-dimensional network.

## Related literature

For background to the chemistry of substituted pyridines, see: Katritzky *et al.* (1996 ▶); Pozharski *et al.* (1997 ▶). For related structures, see: Hemamalini & Fun (2010*a* ▶,*b* ▶). For the conformation of glutaric acid, see: Saraswathi *et al.* (2001 ▶). For details of hydrogen bonding, see: Jeffrey & Saenger (1991 ▶); Jeffrey (1997 ▶); Scheiner (1997 ▶). For hydrogen-bond motifs, see: Bernstein *et al.* (1995 ▶).
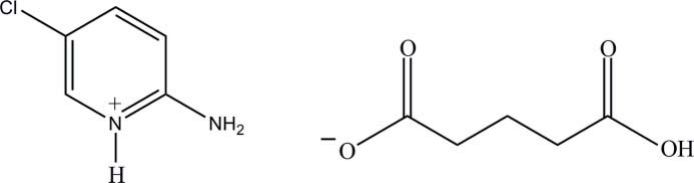


## Experimental

### 

#### Crystal data

C_5_H_6_ClN_2_^+^·C_5_H_7_O_4_^−^
*M* *_r_* = 260.67
Orthorhombic, 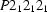

*a* = 5.1970 (14) Å
*b* = 14.509 (4) Å
*c* = 15.970 (5) Å
*V* = 1204.2 (6) Å^3^
*Z* = 4
Mo *K*α radiation
μ = 0.32 mm^−1^
*T* = 296 K
0.31 × 0.13 × 0.07 mm

#### Data collection

Bruker APEXII DUO CCD area-detector diffractometer
Absorption correction: multi-scan (*SADABS*; Bruker, 2009 ▶) *T*_min_ = 0.908, *T*_max_ = 0.979
8054 measured reflections
3346 independent reflections
2007 reflections with *I* > 2σ(*I*)
*R*_int_ = 0.036

#### Refinement

*R*[*F* ^2^ > 2σ(*F* ^2^)] = 0.044
*wR*(*F* ^2^) = 0.111
*S* = 1.01
3346 reflections
162 parameters
H atoms treated by a mixture of independent and constrained refinement
Δρ_max_ = 0.15 e Å^−3^
Δρ_min_ = −0.20 e Å^−3^
Absolute structure: Flack (1983 ▶), 1288 Friedel pairs
Flack parameter: 0.00 (9)

### 

Data collection: *APEX2* (Bruker, 2009 ▶); cell refinement: *SAINT* (Bruker, 2009 ▶); data reduction: *SAINT*; program(s) used to solve structure: *SHELXTL* (Sheldrick, 2008 ▶); program(s) used to refine structure: *SHELXTL*; molecular graphics: *SHELXTL*; software used to prepare material for publication: *SHELXTL* and *PLATON* (Spek, 2009 ▶).

## Supplementary Material

Crystal structure: contains datablocks global, I. DOI: 10.1107/S1600536810027091/is2574sup1.cif

Structure factors: contains datablocks I. DOI: 10.1107/S1600536810027091/is2574Isup2.hkl

Additional supplementary materials:  crystallographic information; 3D view; checkCIF report

## Figures and Tables

**Table 1:** Hydrogen-bond geometry (Å, °)

*D*—H⋯*A*	*D*—H	H⋯*A*	*D*⋯*A*	*D*—H⋯*A*
N1—H1⋯O1^i^	0.86	1.80	2.659 (3)	173
O3—H1*O*3⋯O1^ii^	0.81	1.79	2.598 (2)	173
N2—H1*N*2⋯O2^iii^	0.87 (3)	2.00 (3)	2.848 (3)	163 (3)
N2—H2*N*2⋯O2^i^	0.89 (3)	1.92 (3)	2.808 (3)	173 (2)
C1—H1*A*⋯O4^iv^	0.93	2.44	3.315 (3)	156
C4—H4*A*⋯O4^v^	0.93	2.59	3.396 (3)	145

Symmetry codes: (i) 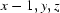; (ii) 
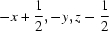
; (iii) 
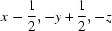
; (iv) 
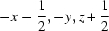
; (v) 
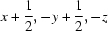
.

**Fig. 1.**
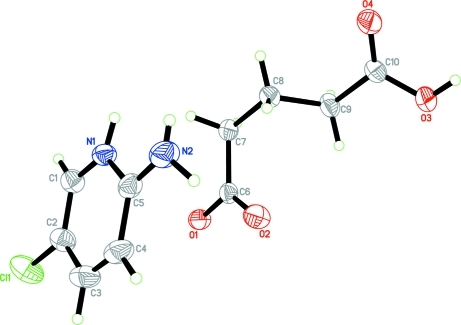
The asymmetric unit of the title compound. Displacement ellipsoids are drawn at the 30% probability level.

**Fig. 2.**
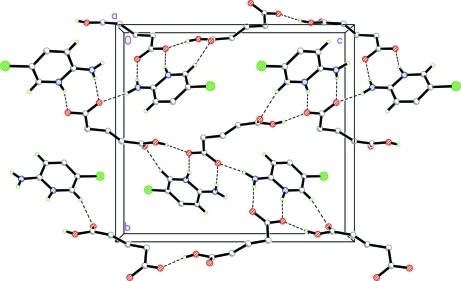
The crystal packing of (I), showing hydrogen-bonded (dashed lines) 2D networks parallel to the bc-plane. H atoms not involved in the intermolecular interactions have been omitted for clarity.

**Fig. 3.**
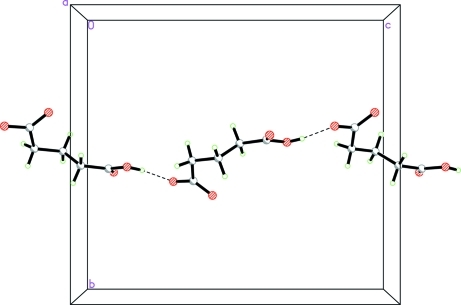
Carboxyl–carboxylate interactions made up of hydrogen glutarate anion.

**Fig. 4.**
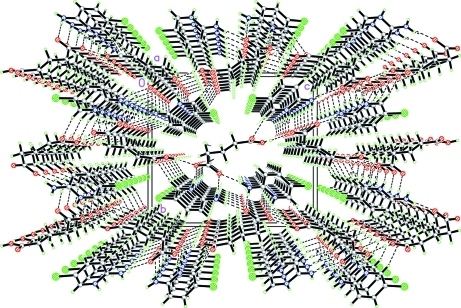
The crystal packing of the title compound (I), showing the stacking of the molecules down the a-axis.

## supplementary crystallographic information

### Comment

Pyridine and its derivatives play an important role in heterocyclic chemistry (Pozharski *et al.*, 1997; Katritzky *et al.*, 1996). They are often involved in hydrogen-bond interactions (Jeffrey & Saenger, 1991; Jeffrey, 1997; Scheiner, 1997).  We have recently reported the crystal structures of 2-amino-5-methylpyridinium 4-carboxybutanoate (Hemamalini & Fun, 2010*a*) and 2-amino-5-bromopyridinium hydrogen glutarate (Hemamalini & Fun, 2010*b*).  In continuation of our studies of pyridinium salts, the crystal structure determination of the title compound has been undertaken.

The asymmetric unit (Fig. 1) contains a 2-amino-5-chloropyridinium cation and a hydrogen glutarate anion. The 2-amino-5-chloropyridinium cation is essentially planar, with a maximum deviation of 0.010 (3) Å for atom C2. The dihedral angle between the pyridine ring and the mean plane formed by the hydrogen glutarate anion is 35.55 (13)°. In the 2-amino-5-chloropyridinium cation, a wide angle [C1—N1—C5= 123.1 (2)°] is subtended at the protonated N1 atom. The backbone conformation of the hydrogen glutarate anion can be described by the two torsion angles C7-C8-C9-C10 of 179.51 (19)° and C6-C7-C8-C9 of 72.4 (3)°. As evident from the torsion angles, the backbone is in a fully extended conformation (Saraswathi *et al.*, 2001) of the two carboxyl groups, one is deprotonated while the other is not.

In the crystal packing, the protonated N1 atom and the 2-amino group (N2) are hydrogen-bonded to the carboxylate oxygen atoms (O1 and O2) via a pair of intermolecular N1—H1···O1 and N2—H1N2···O2 hydrogen bonds, forming a ring motif *R*_2_^2^(8) (Bernstein *et al.*, 1995).  The ion pairs are further connected via N2—H2N2···O2, O3—H1O3···O1 and C4—H4A···O4 (Table 1) hydrogen bonds, forming a two-dimensional network parallel to the *bc* plane (Fig. 2). The hydrogen glutarate anions self-assemble through O3—H1O3···O1 hydrogen bonds, forming one-dimensional supramolecular chains along the *c* axis (Fig. 3). Furthermore, the cations and anions are stacked down along the *a* axis, forming a 3D-network as shown in Fig. 4.  This crystal structure is isomorphous to the crystal structure of 2-amino-5-bromopyridinium hydrogen glutarate (Hemamalini & Fun, 2010*b*).

### Experimental

A hot methanol solution (20 ml) of 2-amino-5-chloropyridine (64 mg, Aldrich) and glutaric acid (66 mg, Merck) were mixed and warmed over a heating magnetic stirrer hotplate for a few minutes. The resulting solution was allowed to cool slowly at room temperature and brown crystals of the title compound appeared after a few days.

### Refinement

Atoms H1N2 and H2N2 were located from a difference Fourier map and were refined freely. The remaining hydrogen atoms were positioned geometrically (N—H = 0.86, O—H = 0.81 and C—H = 0.93 or 0.97 Å) and were refined using a riding model, with *U*_iso_(H) = 1.2*U*_eq_(C, N) or 1.5*U*_eq_(O). 1288 Friedel pairs were used to determine the absolute configuration.

### Crystal data

**Table:**

C_5_H_6_ClN_2_^+^·C_5_H_7_O_4_^−^	*F*(000) = 544
*M**_r_* = 260.67	*D*_x_ = 1.438 Mg m^−^^3^
Orthorhombic, *P*2_1_2_1_2_1_	Mo *K*α radiation, λ = 0.71073 Å
Hall symbol: P 2ac 2ab	Cell parameters from 1567 reflections
*a* = 5.1970 (14) Å	θ = 2.8–25.9°
*b* = 14.509 (4) Å	µ = 0.32 mm^−^^1^
*c* = 15.970 (5) Å	*T* = 296 K
*V* = 1204.2 (6) Å^3^	Plate, brown
*Z* = 4	0.31 × 0.13 × 0.07 mm

### Data collection

**Table:**

Bruker APEXII DUO CCD area-detector diffractometer	3346 independent reflections
Radiation source: fine-focus sealed tube	2007 reflections with *I* > 2σ(*I*)
graphite	*R*_int_ = 0.036
φ and ω scans	θ_max_ = 30.2°, θ_min_ = 1.9°
Absorption correction: multi-scan (*SADABS*; Bruker, 2009)	*h* = −7→7
*T*_min_ = 0.908, *T*_max_ = 0.979	*k* = −20→17
8054 measured reflections	*l* = −22→22

### Refinement

**Table:**

Refinement on *F*^2^	Secondary atom site location: difference Fourier map
Least-squares matrix: full	Hydrogen site location: inferred from neighbouring sites
*R*[*F*^2^ > 2σ(*F*^2^)] = 0.044	H atoms treated by a mixture of independent and constrained refinement
*wR*(*F*^2^) = 0.111	*w* = 1/[σ^2^(*F*_o_^2^) + (0.0443*P*)^2^] where *P* = (*F*_o_^2^ + 2*F*_c_^2^)/3
*S* = 1.01	(Δ/σ)_max_ = 0.001
3346 reflections	Δρ_max_ = 0.15 e Å^−^^3^
162 parameters	Δρ_min_ = −0.20 e Å^−^^3^
0 restraints	Absolute structure: Flack (1983), 1288 Friedel pairs
Primary atom site location: structure-invariant direct methods	Flack parameter: 0.00 (9)

### Special details

**Table:**

Geometry. All s.u.'s (except the s.u. in the dihedral angle between two l.s. planes) are estimated using the full covariance matrix. The cell s.u.'s are taken into account individually in the estimation of s.u.'s in distances, angles and torsion angles; correlations between s.u.'s in cell parameters are only used when they are defined by crystal symmetry. An approximate (isotropic) treatment of cell s.u.'s is used for estimating s.u.'s involving l.s. planes.
Refinement. Refinement of F^2^ against ALL reflections. The weighted R-factor wR and goodness of fit S are based on F^2^, conventional R-factors R are based on F, with F set to zero for negative F^2^. The threshold expression of F^2^ > 2σ(F^2^) is used only for calculating R-factors(gt) etc. and is not relevant to the choice of reflections for refinement. R-factors based on F^2^ are statistically about twice as large as those based on F, and R- factors based on ALL data will be even larger.

### Fractional atomic coordinates and isotropic or equivalent isotropic displacement parameters (Å^2^)

**Table:**

	*x*	*y*	*z*	*U*_iso_*/*U*_eq_	
Cl1	0.34184 (19)	0.28909 (5)	0.39845 (5)	0.0846 (3)	
N1	−0.1438 (4)	0.22907 (12)	0.21798 (11)	0.0464 (5)	
H1	−0.2637	0.1890	0.2106	0.056*	
N2	−0.2316 (5)	0.28468 (18)	0.08678 (14)	0.0633 (6)	
C1	−0.0138 (5)	0.22829 (16)	0.29108 (14)	0.0493 (6)	
H1A	−0.0532	0.1844	0.3315	0.059*	
C2	0.1721 (5)	0.29045 (15)	0.30579 (15)	0.0533 (6)	
C3	0.2266 (6)	0.35662 (17)	0.24403 (18)	0.0628 (7)	
H3A	0.3520	0.4011	0.2536	0.075*	
C4	0.0971 (5)	0.35565 (17)	0.17106 (17)	0.0590 (7)	
H4A	0.1352	0.3992	0.1302	0.071*	
C5	−0.0947 (5)	0.28969 (15)	0.15589 (14)	0.0480 (6)	
O1	0.5116 (3)	0.09526 (11)	0.19901 (9)	0.0523 (4)	
O2	0.4048 (4)	0.14235 (12)	0.07274 (9)	0.0606 (5)	
O3	0.1889 (4)	−0.04241 (12)	−0.15991 (10)	0.0631 (5)	
H1O3	0.1253	−0.0541	−0.2050	0.095*	
O4	−0.1908 (4)	−0.06355 (14)	−0.10194 (11)	0.0672 (5)	
C6	0.3720 (4)	0.09123 (14)	0.13352 (12)	0.0401 (5)	
C7	0.1567 (5)	0.02110 (16)	0.13408 (13)	0.0465 (5)	
H7A	0.0346	0.0381	0.1773	0.056*	
H7B	0.2281	−0.0384	0.1491	0.056*	
C8	0.0127 (5)	0.01093 (16)	0.05204 (13)	0.0476 (6)	
H8A	−0.1445	−0.0236	0.0617	0.057*	
H8B	−0.0348	0.0715	0.0315	0.057*	
C9	0.1709 (5)	−0.03760 (16)	−0.01336 (13)	0.0501 (6)	
H9A	0.3275	−0.0027	−0.0229	0.060*	
H9B	0.2198	−0.0978	0.0078	0.060*	
C10	0.0341 (5)	−0.04956 (14)	−0.09493 (14)	0.0442 (5)	
H1N2	−0.187 (7)	0.3179 (19)	0.0435 (19)	0.076 (9)*	
H2N2	−0.353 (7)	0.242 (2)	0.0786 (17)	0.082 (10)*	

### Atomic displacement parameters (Å^2^)

**Table:**

	*U*^11^	*U*^22^	*U*^33^	*U*^12^	*U*^13^	*U*^23^
Cl1	0.0957 (6)	0.0674 (4)	0.0908 (5)	−0.0065 (5)	−0.0402 (5)	−0.0032 (4)
N1	0.0463 (11)	0.0449 (9)	0.0481 (10)	−0.0088 (10)	0.0025 (9)	0.0045 (8)
N2	0.0720 (16)	0.0697 (15)	0.0482 (13)	−0.0195 (14)	0.0022 (11)	0.0144 (13)
C1	0.0560 (15)	0.0430 (12)	0.0488 (13)	−0.0006 (12)	−0.0008 (12)	0.0034 (11)
C2	0.0518 (14)	0.0437 (12)	0.0642 (14)	0.0028 (13)	−0.0088 (13)	−0.0028 (11)
C3	0.0545 (17)	0.0447 (14)	0.089 (2)	−0.0090 (13)	−0.0021 (15)	0.0035 (14)
C4	0.0580 (17)	0.0483 (13)	0.0706 (17)	−0.0088 (13)	0.0105 (14)	0.0126 (13)
C5	0.0526 (15)	0.0433 (12)	0.0481 (13)	−0.0006 (12)	0.0097 (11)	0.0044 (11)
O1	0.0570 (10)	0.0671 (10)	0.0327 (7)	−0.0186 (9)	−0.0060 (7)	0.0075 (7)
O2	0.0792 (13)	0.0617 (10)	0.0409 (8)	−0.0187 (10)	−0.0131 (8)	0.0155 (8)
O3	0.0598 (11)	0.0885 (12)	0.0409 (9)	−0.0110 (11)	−0.0013 (9)	−0.0122 (8)
O4	0.0462 (10)	0.1013 (14)	0.0540 (10)	−0.0025 (11)	−0.0084 (9)	−0.0141 (10)
C6	0.0429 (13)	0.0446 (11)	0.0328 (10)	0.0016 (11)	0.0009 (10)	−0.0014 (9)
C7	0.0513 (13)	0.0518 (12)	0.0364 (10)	−0.0062 (12)	0.0032 (10)	−0.0012 (9)
C8	0.0453 (13)	0.0537 (13)	0.0438 (12)	−0.0013 (12)	−0.0018 (10)	−0.0068 (10)
C9	0.0490 (13)	0.0598 (15)	0.0417 (12)	0.0065 (13)	−0.0080 (11)	−0.0099 (10)
C10	0.0479 (15)	0.0412 (11)	0.0437 (12)	0.0040 (10)	−0.0075 (11)	−0.0037 (10)

### Geometric parameters (Å, °)

**Table:**

Cl1—C2	1.723 (3)	O2—C6	1.233 (2)
N1—C1	1.349 (3)	O3—C10	1.317 (3)
N1—C5	1.350 (3)	O3—H1O3	0.8102
N1—H1	0.8600	O4—C10	1.192 (3)
N2—C5	1.315 (3)	C6—C7	1.513 (3)
N2—H1N2	0.87 (3)	C7—C8	1.516 (3)
N2—H2N2	0.89 (3)	C7—H7A	0.9700
C1—C2	1.342 (3)	C7—H7B	0.9700
C1—H1A	0.9300	C8—C9	1.504 (3)
C2—C3	1.405 (4)	C8—H8A	0.9700
C3—C4	1.346 (4)	C8—H8B	0.9700
C3—H3A	0.9300	C9—C10	1.494 (3)
C4—C5	1.403 (3)	C9—H9A	0.9700
C4—H4A	0.9300	C9—H9B	0.9700
O1—C6	1.274 (2)		
			
C1—N1—C5	123.1 (2)	O2—C6—C7	120.75 (19)
C1—N1—H1	118.4	O1—C6—C7	116.57 (18)
C5—N1—H1	118.4	C6—C7—C8	115.19 (18)
C5—N2—H1N2	119 (2)	C6—C7—H7A	108.5
C5—N2—H2N2	123.0 (19)	C8—C7—H7A	108.5
H1N2—N2—H2N2	117 (3)	C6—C7—H7B	108.5
C2—C1—N1	120.4 (2)	C8—C7—H7B	108.5
C2—C1—H1A	119.8	H7A—C7—H7B	107.5
N1—C1—H1A	119.8	C9—C8—C7	112.1 (2)
C1—C2—C3	118.8 (2)	C9—C8—H8A	109.2
C1—C2—Cl1	120.74 (19)	C7—C8—H8A	109.2
C3—C2—Cl1	120.5 (2)	C9—C8—H8B	109.2
C4—C3—C2	120.0 (2)	C7—C8—H8B	109.2
C4—C3—H3A	120.0	H8A—C8—H8B	107.9
C2—C3—H3A	120.0	C10—C9—C8	113.5 (2)
C3—C4—C5	120.8 (2)	C10—C9—H9A	108.9
C3—C4—H4A	119.6	C8—C9—H9A	108.9
C5—C4—H4A	119.6	C10—C9—H9B	108.9
N2—C5—N1	118.6 (2)	C8—C9—H9B	108.9
N2—C5—C4	124.5 (2)	H9A—C9—H9B	107.7
N1—C5—C4	116.9 (2)	O4—C10—O3	122.6 (2)
C10—O3—H1O3	115.7	O4—C10—C9	124.6 (2)
O2—C6—O1	122.7 (2)	O3—C10—C9	112.8 (2)
			
C5—N1—C1—C2	1.1 (4)	C3—C4—C5—N2	179.2 (3)
N1—C1—C2—C3	0.6 (4)	C3—C4—C5—N1	0.9 (4)
N1—C1—C2—Cl1	−179.20 (18)	O2—C6—C7—C8	7.5 (3)
C1—C2—C3—C4	−1.4 (4)	O1—C6—C7—C8	−173.7 (2)
Cl1—C2—C3—C4	178.4 (2)	C6—C7—C8—C9	72.4 (3)
C2—C3—C4—C5	0.7 (4)	C7—C8—C9—C10	179.51 (19)
C1—N1—C5—N2	179.8 (2)	C8—C9—C10—O4	−34.9 (3)
C1—N1—C5—C4	−1.8 (3)	C8—C9—C10—O3	144.7 (2)

### Hydrogen-bond geometry (Å, °)

**Table:**

*D*—H···*A*	*D*—H	H···*A*	*D*···*A*	*D*—H···*A*
N1—H1···O1^i^	0.86	1.80	2.659 (3)	173
O3—H1O3···O1^ii^	0.81	1.79	2.598 (2)	173
N2—H1N2···O2^iii^	0.87 (3)	2.00 (3)	2.848 (3)	163 (3)
N2—H2N2···O2^i^	0.89 (3)	1.92 (3)	2.808 (3)	173 (2)
C1—H1A···O4^iv^	0.93	2.44	3.315 (3)	156
C4—H4A···O4^v^	0.93	2.59	3.396 (3)	145

Symmetry codes: (i) *x*−1, *y*, *z*; (ii) −*x*+1/2, −*y*, *z*−1/2; (iii) *x*−1/2, −*y*+1/2, −*z*; (iv) −*x*−1/2, −*y*, *z*+1/2; (v) *x*+1/2, −*y*+1/2, −*z*.

## References

[bb1] Bernstein, J., Davis, R. E., Shimoni, L. & Chang, N.-L. (1995). *Angew. Chem. Int. Ed. Engl.* **34**, 1555–1573.

[bb2] Bruker (2009). *APEX2*, *SAINT* and *SADABS* Bruker AXS Inc., Madison, Wisconsin, USA.

[bb3] Flack, H. D. (1983). *Acta Cryst.* A**39**, 876–881.

[bb4] Hemamalini, M. & Fun, H.-K. (2010*a*). *Acta Cryst.* E**66**, o1841–o1842. 10.1107/S1600536810024451 PMC3006734 21588042

[bb5] Hemamalini, M. & Fun, H.-K. (2010*b*). *Acta Cryst.* E**66**, o1964. 10.1107/S1600536810026280 PMC3007480 21588285

[bb6] Jeffrey, G. A. (1997). *An Introduction to Hydrogen Bonding.* Oxford University Press.

[bb7] Jeffrey, G. A. & Saenger, W. (1991). *Hydrogen Bonding in Biological Structures.* Berlin: Springer.

[bb8] Katritzky, A. R., Rees, C. W. & Scriven, E. F. V. (1996). *Comprehensive Heterocyclic Chemistry II.* Oxford: Pergamon Press.

[bb9] Pozharski, A. F., Soldatenkov, A. T. & Katritzky, A. R. (1997). *Heterocycles in Life and Society.* New York: Wiley.

[bb10] Saraswathi, N. T., Manoj, N. & Vijayan, M. (2001). *Acta Cryst.* B**57**, 366–371. 10.1107/s010876810100218x 11373396

[bb11] Scheiner, S. (1997). *Hydrogen Bonding: A Theoretical Perspective.* Oxford University Press.

[bb12] Sheldrick, G. M. (2008). *Acta Cryst.* A**64**, 112–122. 10.1107/S0108767307043930 18156677

[bb13] Spek, A. L. (2009). *Acta Cryst.* D**65**, 148–155. 10.1107/S090744490804362X PMC2631630 19171970

